# Supplementary material for: The Effect of Prenatal Exposure to Climate Anomaly on Adulthood Cognitive Function and Job Reputation
Source: Int J Environ Res Public Health. 2022 Feb 22;19(5):2523. doi: 10.3390/ijerph19052523 (PMC8909085; doi:10.3390/ijerph19052523)
Supplement: Supplementary file 1 [file ijerph-19-02523-s001.zip › ijerph-1584311-supplementary.pdf]

SUPPLEMENTAL MATERIALS

The Effect of Prenatal Exposure to Climate Anomaly on Adulthood Cognitive Function and Job Reputation

Hong Tang [1], Qian Di* [1,2]

[1] Vanke School of Public Health, Tsinghua University, Beijing, 100084, China
[2] Institute for Healthy China, Tsinghua University, Beijing, 100084, China

Section 1. Additional ENSO indexes

Southern Oscillation Index (SOI): The Southern Oscillation Index (SOI), defined as sea level pressure differences between Tahiti and Darwin, Australia, reflects the activity of El Niño phenomenon. Consecutive negative values of SOI indicate El Niño events; on the contrary, consecutive positive values indicate La Niña events.

Outgoing Longwave Radiation (OLR): Outgoing Longwave Radiation (OLR) is a standardized anomaly index, which reflects the amount of infrared low energy radiation across equatorial areas from 160°E to 160°W longitude. Negative OLR is typical of El Niño episodes. On the contrary, Positive OLR indicates La Niña events.

Table S1 and Figure S1 present summary statistics and time trend for three ENSO indices. The magnitude of these three ENSO indices is different. For example, the mean of OLR (-0.09) was less than zero, while the mean of SST anomalies (0.05) and SOI (0.15) are greater than zero. In addition, the temporal coverage of OLR (1974-1994) was less than that of SST anomalies (1950-1994) and SOI (1951-1994). Our main analysis used SST anomalies for its longest coverage. We used alternative ENSO indicators, such as Southern Oscillation Index (SOI) and Outgoing Longwave Radiation (OLR), as indicators for climate anomalies and repeated main analysis to assess the robustness of our results.

Section 2. The ENSO-teleconnection partition

We used near-surface temperature to define ENSO-teleconnection. We defined a province to be “teleconnected” if the provincially averaged temperature was positively correlated with 2-month-lagged SST anomalies; similarly, we defined a province to be “Weakly affected” if the provincially averaged temperature to be negatively correlated with 2-month-lagged SST anomalies (Figure S9).

Table S1 Descriptive analysis of additional ENSO indexes

ENSO indexes [a]	SST (mean ±SD)	SST 1st (mean ±SD)	SST 2nd (mean ±SD)	SST 3rd (mean ±SD)
**Entire population**	0.05±0.71	0.05±0.81	0.05±0.80	0.04±0.82
**By gender**				
Female	0.05±0.72	0.05±0.82	0.04±0.81	0.04±0.82
Male	0.07±0.70	0.07±0.80	0.07±0.79	0.07±0.81
**By age**				
Age<=45	0.03±0.77	0.03±0.88	0.03±0.87	0.04±0.88
Age>45	0.09±0.60	0.09±0.69	0.07±0.69	0.06±0.71
**By smoking status**				
Smokers	0.08±0.69	0.09±0.79	0.07±0.78	0.05±0.80
Non smokers	0.05±0.72	0.05±0.82	0.04±0.81	0.04±0.82
**By income level**				
Income level<=2	0.04±0.72	0.05±0.81	0.03±0.81	0.03±0.82
Income level>2	0.06±0.71	0.06±0.81	0.06±0.80	0.06±0.81
**By GDP per capita (US dollar)**				
GDP per capita<= 1880	0.05±0.72	0.05±0.82	0.04±0.82	0.03±0.83
GDP per capita > 1880	0.07±0.70	0.06±0.80	0.06±0.78	0.06±0.80
**By region associated with ENSO**				
Weakly affected	0.06±0.71	0.05±0.81	0.05±0.80	0.05±0.82
Strong teleconnected	0.03±0.71	0.04±0.83	0.03±0.81	0.03±0.71
**ENSO indexes [a]**	**SOI (mean ±SD)**	**SOI 1st (mean ±SD)**	**SOI 2nd (mean ±SD)**	**SOI 3rd (mean ±SD)**
**Entire population**	0.15±0.67	0.15±0.78	0.15±0.79	0.16±0.79
**By gender**				
Female	0.17±0.67	0.16±0.79	0.16±0.79	0.17±0.79
Male	0.13±0.66	0.13±0.77	0.13±0.78	0.12±0.78
**By age**				
Age<=45	0.11±0.74	0.11±0.86	0.11±0.87	0.10±0.87
Age>45	0.24±0.51	0.23±0.62	0.24±0.61	0.25±0.62
**By smoking status**				
Smokers	0.15±0.64	0.14±0.75	0.16±0.75	0.15±0.75
Non-smokers	0.16±0.67	0.15±0.79	0.15±0.79	0.16±0.79
**By income level**				
Income level<=2	0.17±0.66	0.16±0.77	0.17±0.78	0.17±0.79
Income level>2	0.15±0.67	0.15±0.79	0.15±0.79	0.15±0.79
**By GDP per capita (US dollar)**				
GDP per capita<= 1880	0.16±0.68	0.15±0.79	0.16±0.80	0.17±0.80
GDP per capita > 1880	0.15±0.65	0.15±0.77	0.15±0.76	0.14±0.78
**By region associated with ENSO**				
Weakly affected	0.15±0.67	0.15±0.78	0.15±0.78	0.15±0.79
Strong teleconnected	0.17±0.67	0.16±0.80	0.17±0.80	0.18±0.78
**ENSO indexes [a]**	**OLR (mean ±SD)**	**OLR 1st (mean ±SD)**	**OLR 2nd (mean ±SD)**	**OLR 3rd (mean ±SD)**
**Entire population**	-0.09±0.76	-0.07±0.86	-0.08±0.85	-0.11±0.85
**By gender**				
Female	-0.08±0.75	-0.06±0.86	-0.07±0.85	-0.10±0.85
Male	-0.13±0.77	-0.11±0.87	-0.11±0.86	-0.14±0.86
**By age**				
Age<=45	-0.09±0.76	-0.07±0.86	-0.08±0.85	-0.11±0.85
Age>45	NA	NA	NA	NA
**By smoking status**				
Smokers	-0.07±0.78	-0.06±0.87	-0.09±0.88	-0.05±0.84
Non-smokers	-0.09±0.76	-0.07±0.86	-0.08±0.85	-0.11±0.85
**By income level**				
Income level<=2	-0.08±0.76	-0.07±0.85	-0.07±0.86	-0.09±0.87
Income level>2	-0.09±0.74	-0.05±0.85	-0.08±0.84	-0.11±0.83
**By GDP per capita (US dollar)**				
GDP per capita<= 1880	-0.09±0.77	-0.08±0.88	-0.09±0.86	-0.10±0.86
GDP per capita > 1880	-0.09±0.74	-0.06±0.83	-0.07±0.83	-0.11±0.85
**By region associated with ENSO**				
Weakly affected	-0.10±0.76	-0.07±0.86	-0.09±0.86	-0.12±0.86
Strong teleconnected	-0.06±0.75	-0.08±0.85	-0.05±0.84	-0.04±0.83

Note: **a**: We used 2-month-lagged SST (sea surface temperature) anomalies in the main text, and SOI (Southern Oscillation Index) and OLR (Outgoing Longwave Radiation) with 2-month lag in the supplementary material. The definition and categorization of other variables were specified in the footnote of Table 1.

Table S2 Economic loss due to prenatal exposure to abnormal climate mediated by math test score

Step	Model	Estimate (95%CI)
**Step1: Climate-cognition association [a]**	Change in math test score (SST anomalies increasing 1 ℃ from 0 ℃) (point)	-0.09 (-0.11, -0.07)
	Change in math test score (SST anomalies decreasing 1 ℃ from 0 ℃) (point)	-0.30 (-0.34, -0.26)
**Step 2: Cognition-income association [b]**	Percent change of income level with 1 point increment of math test score	11.54% (10.70%, 12.39%)
	Absolute change of income level with 1 point increment of math test score (US dollar)	230.53 (213.64, 247.55)
**Step 3: Income loss due to climate by person by year [c]**	Income loss due to lowered math test score causing by abnormal climate (per person each year) (SST anomalies increasing 1 ℃ from 0 ℃) (US dollar)	-20.75 (-25.36, -16.14)
	Income loss due to lowered math test score causing by abnormal climate (per person each year) (SST anomalies decreasing 1 ℃ from 0 ℃) (US dollar)	-69.16 (-78.38, -59.94)
**Step 4: Total income loss [d]**	Economic cost of intellectual impairment causing by unfavored climate (SST anomalies increasing 1 ℃ from 0 ℃) (billion US dollar)	-0.33 (-0.40, -0.25)
	Economic cost of intellectual impairment causing by unfavored climate (SST anomalies decreasing 1 ℃ from 0 ℃) (billion US dollar)	-1.09 (-1.23, -0.94)

Note: **a**: This model used math test score as the dependent variable and SST anomalies as the independent variable, and adjusted for fixed effects of age, smoking status, gender, physical exercise frequency, the wave of follow up, and a random intercept for individual, but this model did not adjust for fixed effect of income. **b**: This model used log-transformed income as the dependent variable, and math test score as the independent variable, and adjusted for fixed effect of age, smoking status, gender, physical exercise frequency, the wave of follow up, and random intercept for individual. Absolute change of income associated with an increment change of math test score = percent change of income associated with an increment change of math test score *average income level (1997.27 USD); **c**: Income loss associated with prenatal exposure to climate anomalies = cognitive change associated with prenatal exposure to climate anomalies * income change associated with cognitive change. **d**: This calculation was based on China’s population and birth rate for the year 2018.

Table S3 The distribution of salary loss for lowered math test score causing by unfavored climate condition in different regions of mainland China

Region	Population of newborn (1000 persons)	Income loss (SST anomalies increasing 1 °C from 0 °C) (in 1000 US dollar) (95%CI)	Income loss (SST anomalies decreasing 1 °C from 0 °C) (in 1000 US dollar) (95%CI)
**Beijing**	177.49	3682.91 (4501.14, 2864.68)	12275.18 (13911.63, 10638.73)
**Tianjin**	104.05	2159.08 (2638.76, 1679.40)	7196.24 (8155.60, 6236.88)
**Hebei Province**	850.81	17654.22 (21576.43, 13732.00)	58841.72 (66686.14, 50997.29)
**Shanxi Province**	358.04	7429.40 (9079.98, 5778.82)	24762.28 (28063.44, 21461.12)
**Nei Monggol Autonomous Region**	211.59	4390.47 (5365.90, 3415.05)	14633.50 (16584.35, 12682.64)
**Liaoning Province**	278.54	5779.71 (7063.78, 4495.64)	19263.83 (21831.97, 16695.69)
**Jilin Province**	179.00	3714.35 (4539.56, 2889.14)	12379.97 (14030.40, 10729.55)
**Heilongjiang Province**	225.63	4681.73 (5721.86, 3641.59)	15604.25 (17684.52, 13523.99)
**Shanghai**	174.53	3621.46 (4426.03, 2816.88)	12070.36 (13679.50, 10461.21)
**Jiangsu Province**	750.35	15569.83 (19028.96, 12110.70)	51894.43 (58812.68, 44976.17)
**Zhejiang Province**	632.22	13118.51 (16033.03, 10203.99)	43724.16 (49553.20, 37895.11)
**Anhui Province**	784.81	16284.77 (19902.74, 12666.81)	54277.35 (61513.28, 47041.42)
**Fujian Province**	520.21	10794.40 (13192.58, 8396.22)	35977.86 (40774.22, 31181.51)
**Jiangxi Province**	624.23	12952.70 (15830.38, 10075.01)	43171.50 (48926.87, 37416.13)
**Shandong Province**	1332.23	27643.82 (33785.41, 21502.23)	92137.18 (104420.36,79854.00)
**Henan Province**	1125.71	23358.40 (28547.90, 18168.89)	77853.83 (88232.84, 67474.82)
**Hubei Province**	682.82	14168.55 (17316.36, 11020.74)	47223.96 (53519.57, 40928.34)
**Hunan Province**	840.99	17450.50 (21327.46, 13573.55)	58162.74 (65916.65, 50408.83)
**Guangdong Province**	1451.15	30111.43 (36801.25, 23421.62)	100361.77 (113741.40, 86982.13)
**the Guangxi Zhuang Autonomous Region**	695.55	14432.69 (17639.18, 11226.20)	48104.32 (54517.30, 41691.34)
**Hainan Province**	135.24	2806.30 (3429.77, 2182.83)	9353.42 (10600.36, 8106.48)
**Chongqing**	341.84	7093.19 (8669.07, 5517.30)	23641.68 (26793.45, 20489.91)
**Sichuan Province**	921.68	19124.87 (23373.82, 14875.92)	63743.42 (72241.32, 55245.53)
**Guizhou Province**	500.40	10383.30 (12690.14,8076.46)	34607.66 (39221.35, 29993.98)
**Yunnan Province**	637.08	13219.35 (16156.27, 10282.42)	44060.25 (49934.10, 38186.40)
**Tibet Autonomous Region**	52.36	1086.40 (1327.77, 845.04)	3621.00 (4103.73, 3138.27)

**shaanxi Province**	412.29	8554.99 (10455.64, 6654.34)	28513.89 (32315.20, 24712.59)
**Gansu Province**	291.92	6057.25 (7402.99, 4711.52)	20188.90 (22880.37, 17497.44)
**Qinghai Province**	86.29	1790.50 (2188.30, 1392.71)	5967.77 (6763.36, 5172.18)
**the Ningxia Hui Autonomous Region**	91.64	1901.56 (2324.03, 1479.10)	6337.93 (7182.87, 5493.00)
**the Xinjiang Uygur Autonomous Region**	265.86	5516.60 (6742.22, 4290.99)	18386.90 (20838.13, 15935.67)
**Total**	15736.54	326533.24 (399078.70, 253987.78)	1088339.23 (1233430.15, 943248.32)

Table S4 Economic loss due to prenatal exposure to abnormal climate mediated by word test score

Step	Model	Estimate (95%CI)
**Step1: Climate-cognition association**	Change in word test score (SST anomalies increasing 1 ℃ from 0 ℃) (point)	-0.08 (-0.12, -0.05)
	Change in word test score (SST anomalies decreasing 1 ℃ from 0 ℃) (point)	-0.26 (-0.33, -0.19)
**Step 2: Cognition-income association**	Percent change of income level with 1 point increment of word test score	5.97% (5.46%, 6.47%)
	Absolute change of income level with 1 point increment of word test score (US dollar)	119.20 (109.14, 129.30)
**Step 3: Income loss due to climate by person by year**	Income loss due to lowered word test score causing by abnormal climate (per person each year) (SST anomalies increasing 1 ℃ from 0 ℃) (US dollar)	-9.54 (-14.30, -5.96)
	Income loss due to lowered word test score causing by abnormal climate (per person each year) (SST anomalies decreasing 1 ℃ from 0 ℃) (US dollar)	-30.99 (-39.34, -22.65)
**Step 4: Total income loss**	Economic cost of intellectual impairment causing by unfavored climate (SST anomalies increasing 1 ℃ from 0 ℃) (billion dollar)	-0.15 (-0.23, -0.09)
	Economic cost of intellectual impairment causing by unfavored climate (SST anomalies decreasing 1 ℃ from 0 ℃) (billion dollar)	-0.49 (-0.62, -0.36)

Note: we repeated the same steps as in Table S2 and replaced math test score with the word test score.

Table S5 The distribution of salary loss for lowered word test score causing by unfavored climate condition in different regions of mainland China

Region	Population of newborn (1000 persons)	Income loss (SST anomalies increasing 1 ℃ from 0 ℃) (in 1000 US dollar) (95%CI)	Income loss (SST anomalies decreasing 1 ℃ from 0 ℃) (in 1000 US dollar) (95%CI)
**Beijing**	177.49	1693.25 (2538.10, 1057.84)	5500.40 (6982.44, 4020.14)
**Tianjin**	104.05	992.66 (1487.94, 620.15)	3224.57 (4093.41, 2356.78)
**Hebei Province**	850.81	8116.69 (12166.52, 5070.80)	26366.47 (33470.69, 19270.75)
**Shanxi Province**	358.04	3415.73 (5120.02, 2133.94)	11095.76 (14085.43, 8109.68)
**Nei Monggol Autonomous Region**	211.59	2018.56 (3025.72, 1261.07)	6557.14 (8323.91, 4792.49)
**Liaoning Province**	278.54	2657.27 (3983.12, 1660.10)	8631.96 (10957.77, 6308.93)
**Jilin Province**	179.00	1707.71 (2559.77, 1066.87)	5547.36 (7042.05, 4054.46)
**Heilongjiang Province**	225.63	2152.47 (3226.44, 1344.73)	6992.13 (8876.10, 5110.42)
**Shanghai**	174.53	1665.00 (2495.75, 1040.19)	5408.62 (6865.93, 3953.06)
**Jiangsu Province**	750.35	7158.37 (10730.05, 4472.11)	23253.45 (29518.89, 16995.50)
**Zhejiang Province**	632.22	6031.35 (9040.71, 3768.02)	19592.42 (24871.43, 14319.72)
**Anhui Province**	784.81	7487.07 (11222.76, 4677.46)	24321.21 (30874.36, 17775.91)
**Fujian Province**	520.21	4962.82 (7439.03, 3100.46)	16121.37 (20465.14, 11782.80)
**Jiangxi Province**	624.23	5955.12 (8926.44, 3720.39)	19344.78 (24557.07, 14138.73)
**Shandong Province**	1332.23	12709.50 (19050.92, 7940.10)	41285.88 (52410.01, 30175.06)
**Henan Province**	1125.71	10739.24 (16097.60, 6709.21)	34885.63 (44285.27, 25497.24)
**Hubei Province**	682.82	6514.12 (9764.35, 4069.62)	21160.65 (26862.21, 15465.91)
**Hunan Province**	840.99	8023.03 (12026.13, 5012.29)	26062.22 (33084.47, 19048.38)
**Guangdong Province**	1451.15	13844.00 (20751.49, 8648.87)	44971.24 (57088.37, 32868.62)
**the Guangxi Zhuang Autonomous Region**	695.55	6635.56 (9946.38, 4145.49)	21555.13 (27362.98, 15754.23)
**Hainan Province**	135.24	1290.22 (1933.98, 806.05)	4191.19 (5320.47, 3063.26)
**Chongqing**	341.84	3261.16 (4888.32, 2037.37)	10593.63 (13448.00, 7742.69)
**Sichuan Province**	921.68	8792.83 (13180.03, 5493.22)	28562.88 (36258.91, 20876.06)
**Guizhou Province**	500.40	4773.82 (7155.72, 2982.38)	15507.40 (19685.74, 11334.06)
**Yunnan Province**	637.08	6077.71 (9110.20, 3796.98)	19743.02 (25062.61, 14429.79)
**Tibet Autonomous Region**	52.36	499.48 (748.70, 312.05)	1622.54 (2059.72, 1185.88)
**shaanxi province**	412.29	3933.24 (5895.73, 2457.24)	12776.83 (16219.44, 9338.34)
**Gansu Province**	291.92	2784.88 (4174.40, 1739.82)	9046.47 (11483.97, 6611.90)

**Qinghai Province**	86.29	823.20 (1233.94, 514.28)	2674.11 (3394.62, 1954.45)
**the Ningxia Hui Autonomous Region**	91.64	874.26 (1310.47, 546.18)	2839.97 (3605.18, 2075.68)
**the Xinjiang Uygur Autonomous Region**	265.86	2536.31 (3801.80, 1584.53)	8239.01 (10458.94, 6021.74)
**Total**	15736.54	150126.61 (225032.55, 93789.79)	487675.43 (619075.55, 356432.67)

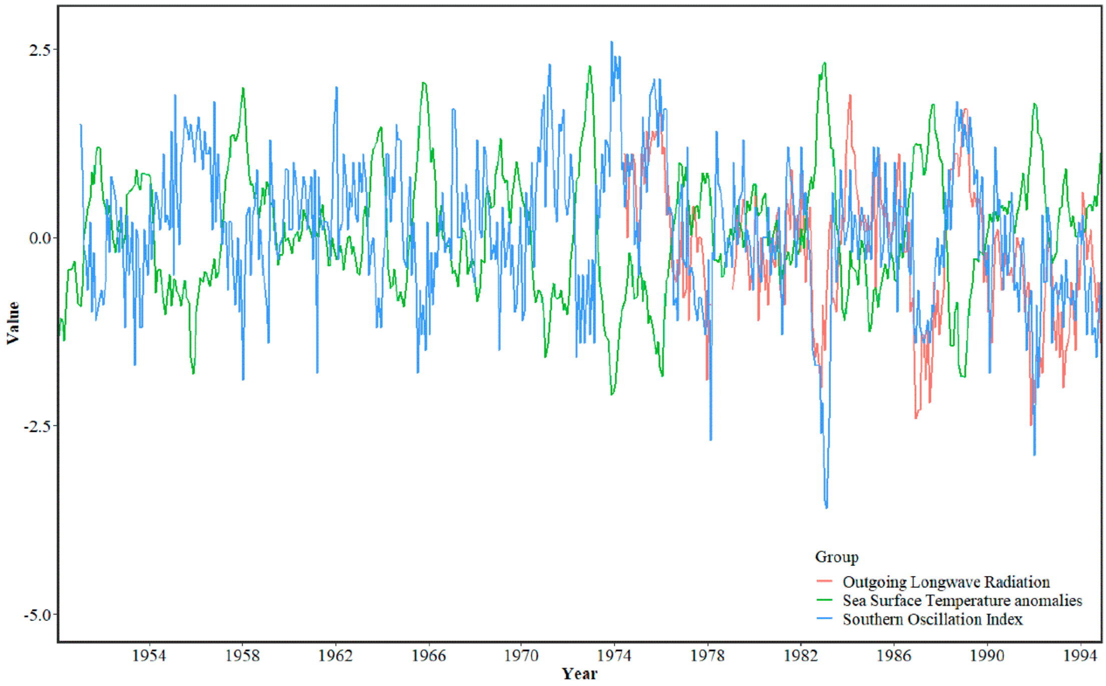

Figure S1 Monthly Sea Surface Temperature anomalies in the Niño 3.4 region (SST), Outgoing Longwave Radiation, and Southern Oscillation Index, 1950-1994

Note: SST anomalies, outgoing longwave radiation and southern oscillation index have different temporal coverages. Source: https://www.cpc.ncep.noaa.gov/data/indices

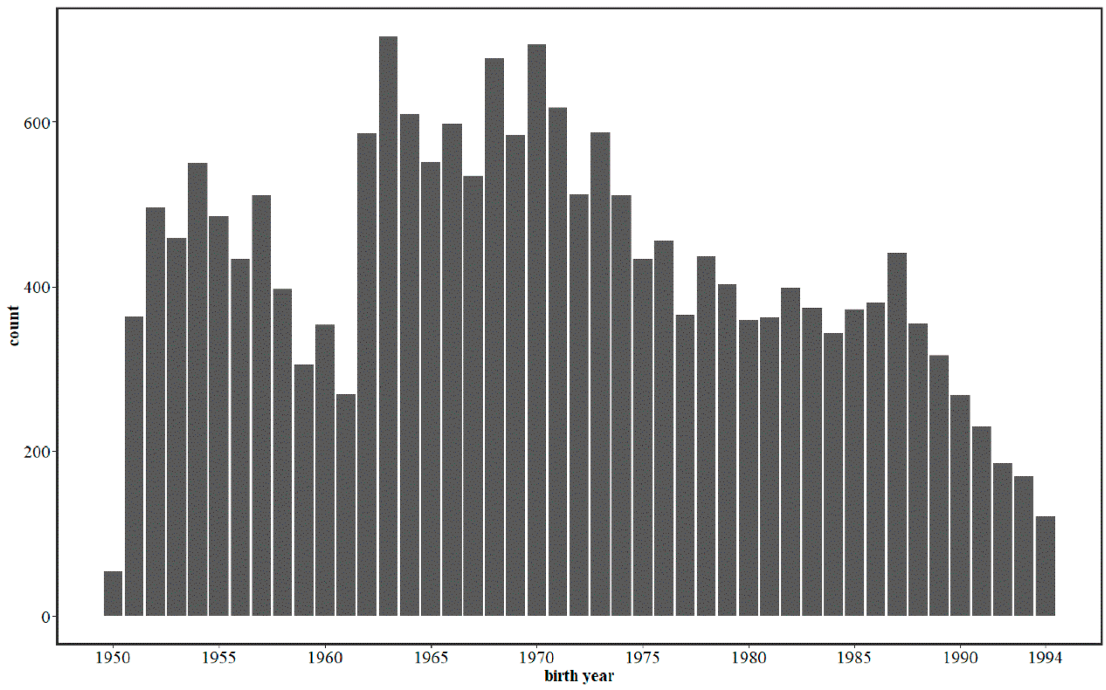

Figure S2 Distribution of birth years of CFPS individuals

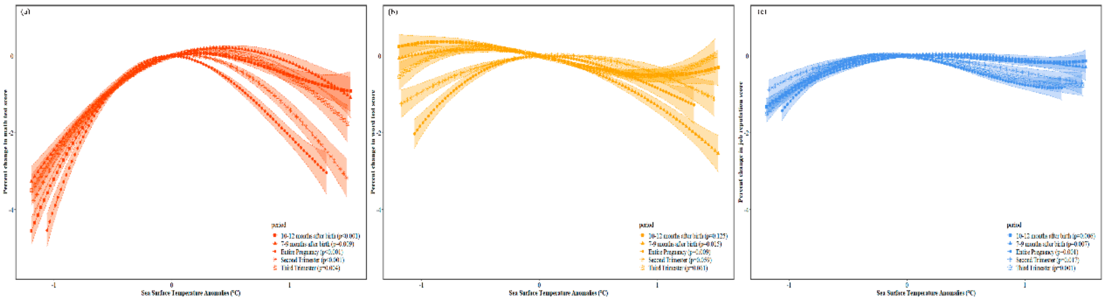

Figure S3 Dose-response relationship between adulthood cognitive function, job reputation and climate anomalies during different gestational periods and periods after birth.

Note: We assessed dose-response relationship with between SST anomalies and math test score (a), word test score (b), and job reputation (c). Dependent variables were math test score, word test score and job reputation score; independent variables were SST anomalies at different time periods (entire pregnancy, second trimester, third trimester, 7-9 months after birth and 10-12 months after birth). Models were adjusted for fixed-effect of age, gender, log-transformed income, smoking status, and physical exercise frequency, and a random effect for individual. To account for any nonlinear relationship, we put B-spline with 3 degrees of freedom as the smooth function on SST anomalies in the mixed-effect model. We converted changes in cognitive test score/job reputation score into percent change by dividing the mean value. Percent changes in cognitive test score and job reputation score were centered at SST anomalies = 0 °C. Dose-response curves outside 5th and 95th percentiles of SST anomalies were trimmed.

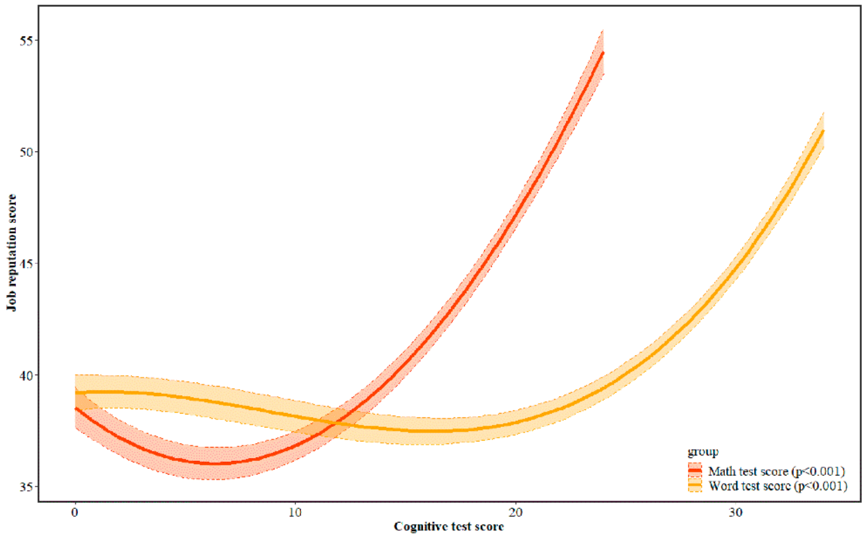

Figure S4 The association between cognitive ability and job reputation

Note: We used two mixed-effect models with random effect on individual to estimate the association between cognitive ability (math test score, word test score) and job reputation. In the two models, the dependent variable was job reputation score; independent variables were math test and word test score, respectively. The two models were adjusted for fixed-effect of age, gender, log-transformed income, smoking status, and physical exercise frequency, and random effect for individual. To account for any nonlinear relationship, we put B-spline with 3 degrees of freedom as the smooth function on independent variables.

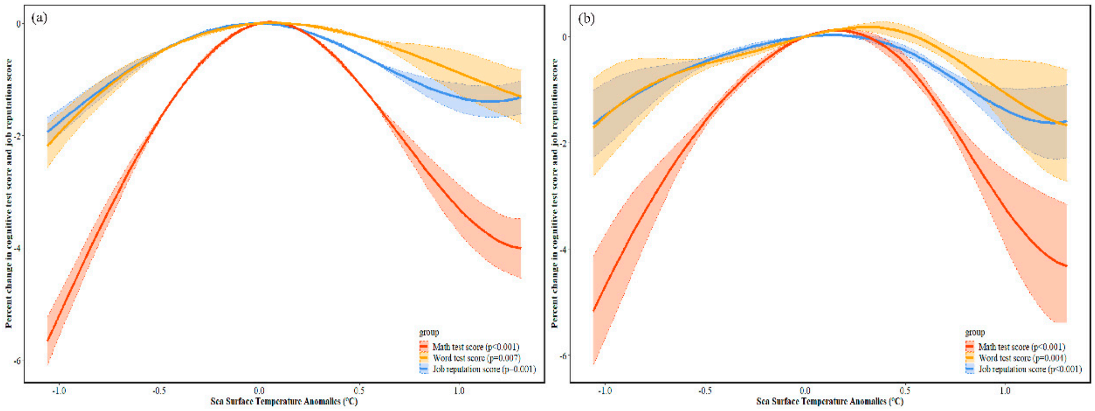

Figure S5 Dose-response relationship between adulthood cognitive function, job reputation and prenatal climate anomaly in entire pregnancy (a) df=4 (b) df=5

Note: We repeated the same process as in Figure 1 but changed the degree of freedom of smooth function into 4 and 5.

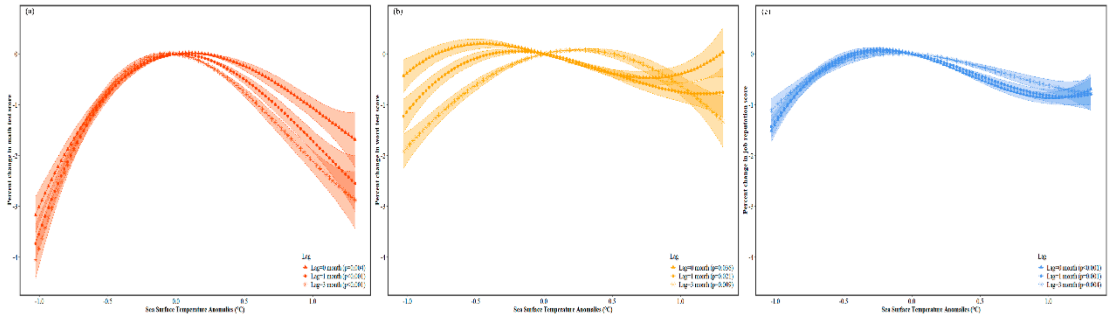

Figure S6 Dose-response relationship between adulthood cognitive function, job reputation and climate anomaly with different time lags in entire pregnancy (a) math test score (b) word test score (c) job reputation score

Note: We repeated the same process as in Figure 1 but used 0-month-lagged, 1-month-lagged, and 3-month-lagged SST anomalies.

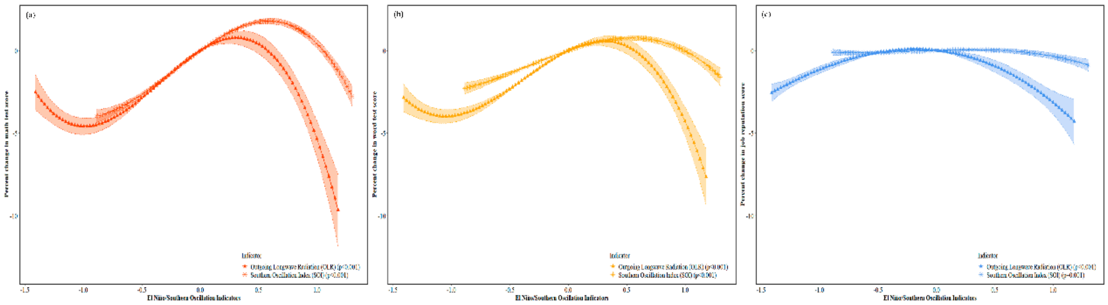

Figure S7 Dose-response relationship between different climate anomaly indicators and adulthood cognitive function and job reputation in entire pregnancy (a) math test score (b) word test score (c) job reputation score

Note: We repeated the same process as in Figure 1 but used other indictors of ENSO (Southern Oscillation Index and Outgoing Longwave Radiation).

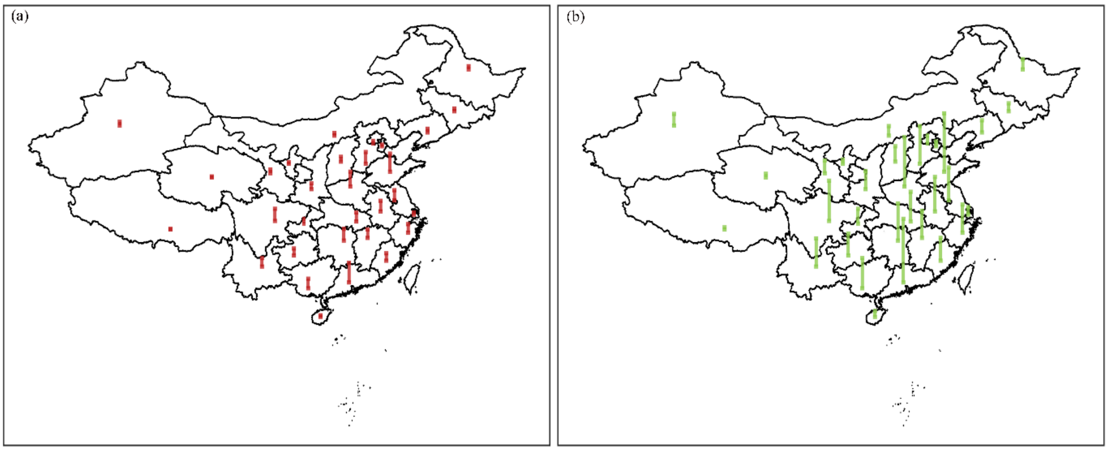

Figure S8 Economic loss for lowed word test score associated prenatal exposure to climate anomaly in different regions of China (a) SST anomalies increasing 1 ℃ from 0 ℃ (b) SST anomalies decreasing 1 ℃ from 0 ℃

Note: We repeated the same process as in Figure 4 but replaced math test score with word test score.

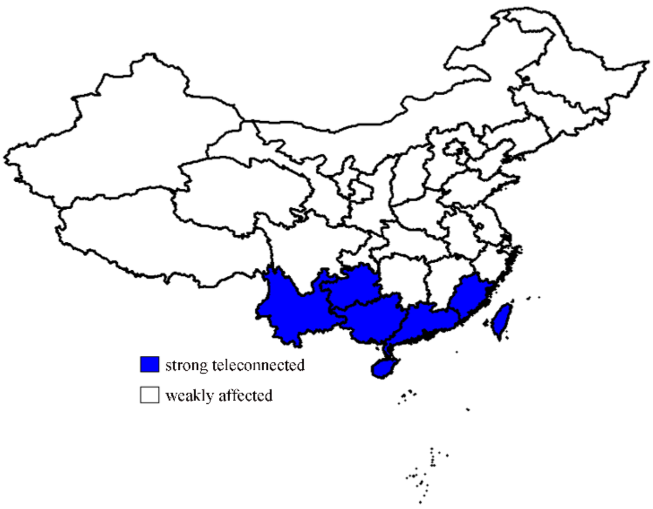

Figure S9 ENSO teleconnection partition for China.

Note: we defined teleconnected-regions (coded as blue) based on the correlation between 2-month-lagged SST anomalies and provincial temperature.
